# Changes in the Spinal Neural Circuits are Dependent on the Movement Speed of the Visuomotor Task

**DOI:** 10.3389/fnhum.2015.00667

**Published:** 2015-12-15

**Authors:** Shinji Kubota, Masato Hirano, Yoshiki Koizume, Shigeo Tanabe, Kozo Funase

**Affiliations:** ^1^Human Motor Control Laboratory, Department of Human Sciences, Graduate School of Integrated Arts and Sciences, Hiroshima UniversityHiroshima, Japan; ^2^Research Fellow of the Japan Society for the Promotion of ScienceTokyo, Japan; ^3^Faculty of Rehabilitation, School of Health Sciences, Fujita Health UniversityAichi, Japan

**Keywords:** movement speed, presynaptic inhibition, reciprocal Ia inhibition, visuomotor task, spinal plasticity

## Abstract

Previous studies have shown that spinal neural circuits are modulated by motor skill training. However, the effects of task movement speed on changes in spinal neural circuits have not been clarified. The aim of this research was to investigate whether spinal neural circuits were affected by task movement speed. Thirty-eight healthy subjects participated in this study. In experiment 1, the effects of task movement speed on the spinal neural circuits were examined. Eighteen subjects performed a visuomotor task involving ankle muscle slow (nine subjects) or fast (nine subjects) movement speed. Another nine subjects performed a non-visuomotor task (controls) in fast movement speed. The motor task training lasted for 20 min. The amounts of D1 inhibition and reciprocal Ia inhibition were measured using H-relfex condition-test paradigm and recorded before, and at 5, 15, and 30 min after the training session. In experiment 2, using transcranial magnetic stimulation (TMS), the effects of corticospinal descending inputs on the presynaptic inhibitory pathway were examined before and after performing either a visuomotor (eight subjects) or a control task (eight subjects). All measurements were taken under resting conditions. The amount of D1 inhibition increased after the visuomotor task irrespective of movement speed (*P* < 0.01). The amount of reciprocal Ia inhibition increased with fast movement speed conditioning (*P* < 0.01), but was unchanged by slow movement speed conditioning. These changes lasted up to 15 min in D1 inhibition and 5 min in reciprocal Ia inhibition after the training session. The control task did not induce changes in D1 inhibition and reciprocal Ia inhibition. The TMS conditioned inhibitory effects of presynaptic inhibitory pathways decreased following visuomotor tasks (*P* < 0.01). The size of test H-reflex was almost the same size throughout experiments. The results suggest that supraspinal descending inputs for controlling joint movement are responsible for changes in the spinal neural circuits, and that task movement speed is one of the critical factors for inducing plastic changes in reciprocal Ia inhibition.

## Introduction

Plastic changes in cortical areas induced by motor skill training have been investigated, and results suggest they are related to the acquisition of motor skills (Karni et al., 1995; Pascual-Leone et al., 1995; Muellbacher et al., 2001, 2002; Perez et al., 2004). It was reported that active-dependent plasticity develops not only at cortical levels but also at the spinal level (Wolpaw, 2007). In support of this concept, previous studies showed that motor skill training could induce reorganization of the spinal cord, which might also account for the improvement of motor performance (Perez et al., 2005; Mazzocchio et al., 2006; Meunier et al., 2007; Roche et al., 2011).

The spinal cord receives sensory inputs arising from cutaneous and proprioceptive receptors. As sensory inputs to the spinal cord vary depending on motor tasks (e.g., movement type, task difficulty level, and movement speed; Popple and Bowman, 1970; Kakuda et al., 1997; Bosco and Poppele, 1999; Jones et al., 2001), and are likely to influence the activity level of muscle during voluntary movement (Nielsen and Sinkjaer, 2002; Seki et al., 2003), modification of sensory signals appears to be a critical factor in executing skilled motor tasks (Doemges and Rack, 1992; Dun et al., 2007). Previous studies have shown that presynaptic inhibition is one of the key mechanisms in the spinal cord to regulate these sensory signals (Seki et al., 2003; Seki and Fetz, 2012). Presynaptic inhibition produces primary afferent depolarization (PAD) of sensory afferent fibers and is caused by activation of GABAergic interneurons (PAD interneurons) forming axo-axonic contacts with sensory afferent terminals, which lead to a reduction in the release of neurotransmitters from the sensory afferents (Rudomín, 2009). Changes in the sensory inputs at preneuron levels contribute to the control of the spinal reflexes, such as the stretch reflex and/or cutaneous reflex (Sinkjaer and Hayashi, 1989; Bawa and Sinkjaer, 1999).

In humans, spinal cord plasticity has been inferred from modification in the size of the H-reflex that is the electrical analog of the monosynaptic stretch reflex (Thompson and Wolpaw, 2014), and presynaptic inhibition has been suggested to be related to changes in the H-reflex following motor skill training (Perez et al., 2005; Roche et al., 2011). Although several studies showed that presynaptic inhibition is modulated by motor skill training, the effects of task movement speed on the changes in the presynaptic inhibition have not been clarified. Based on the fact that muscle spindle is sensitive to the velocity of muscle stretch (Popple and Bowman, 1970; Cronin et al., 2009), it is conceivable that presynaptic inhibition of primary sensory fibers of the muscle spindle (group Ia afferent) is differently modulated, dependent on the task movement speed. Moreover, with increments of task movement speed, agonist/antagonist muscles have to switch their activity as quickly as possible to execute an alternating joint movement. Hence, in this situation it may be necessary to facilitate the spinal reciprocal Ia inhibitory circuit, because this circuit coordinates the contraction and relaxation of opposing sets of muscles (Geertsen et al., 2011). Therefore, it appears reasonable to assume that the plastic changes in these spinal neural circuits are dependent on the task movement speed. We hypothesize that the presynaptic inhibition and reciprocal Ia inhibition will be increased following motor skill training performed at a fast movement speed.

The aim of this research is to investigate whether changes in the spinal neural circuits are affected by task movement speed. To address this question, we examined the amount of the presynaptic inhibition and reciprocal Ia inhibition before and after the visuomotor task that was set to either slow or fast movement speed. It has also been suggested that descending inputs delivered via the corticospinal tract influence the activity levels of interneurons constituting these spinal neural circuits (Jankowska, 1992). Therefore, the corticospinal descending inputs may play an important role in driving the plastic changes in the spinal neural circuits observed following the skilled motor task. Hence, in order to clarify responsible mechanisms involved in the modification of spinal neural circuits following skilled motor task, we also examined the effects of corticospinal descending inputs on the presynaptic inhibitory pathway using transcranial magnetic stimulation (TMS) conditioning techniques (Meunier and Pierrot-Deseilligny, 1998).

## Materials and Methods

### Subjects

Thirty-eight healthy subjects [age, 22.8 ± 2.7 years; mean ± standard deviation (SD)] participated in this study after providing written informed consent. Baseline characteristics of participants are shown in Table 1. All experimental procedures were carried out in accordance with the Declaration of Helsinki and were approved by the Human Ethics Committee of the Graduate School of Integrated Arts and Sciences of Hiroshima University.

**Table 1:** **Baseline characteristics of subjects (mean ± SD)**.

	Experiment 1			Experiment 2	
	Slow speed group	Fast speed group	Control group	Visuomotor group	Non-visuomotor group
Age	23.77 ± 2.86	23.0 ± 4.24	22.55 ± 2.45	22.37 ± 1.59	23.75 ± 2.25
Sex (Male/Female)	6/3	6/3	7/2	6/2	6/2
SOL Mmax (mV)	13.54 ± 3.31	14.75 ± 6.42	14.05 ± 4.32	16.42 ± 5.53	14.71 ± 4.23
Hmax/Mmax	0.53 ± 0.18	0.60 ± 0.19	0.59 ± 0.17	0.48 ± 0.13	0.48 ± 0.17
Active MT (% of SO)	—	—	—	46.5 ± 10.11	48.25 ± 6.18
Stimulus intensity (% of SO)	—	—	—	38.37 ± 7.11	40.87 ± 4.67

*Mmax, maximum amplitude of M-wave; Hmax, maximum amplitude of H-reflex; MT, motor threshold; SO, stimulator output; SOL, soleus*.

### EMG Recording

Subjects were seated in an armchair with the examined leg semi-flexed at the hip (120°) and the knee (120°), and plantar-flexed at the ankle (110°). The right lower leg was secured with an ankle-foot orthosis brace. All experimental measurements were taken while in a resting condition.

Electromyographic (EMG) activity was recorded with bipolar surface electrodes (9-mm diameter Ag/AgCl surface cup electrodes; 20 mm distance between electrodes) placed on the right soleus (SOL) and tibialis anterior (TA) muscle belly. Raw EMG signals were amplified at 1000 times and band-pass filtered between 5 and 3000 Hz, using an amplifier (model 7S12; NEC San-ei Co., Ltd., Tokyo, Japan). The EMG signals were digitized by an analog/digital (A/D) converter with a sampling rate of 10 kHz (PowerLab System Scope version 3.7.6; AD Instruments Pty. Ltd., Dunedin, New Zealand) and stored on a computer for subsequent analyses. The recording period was 200 ms including the pre-stimulus period of 50 ms.

SOL H-reflex and M-wave were evoked by stimulating the posterior tibial nerve through a monopolar stimulating electrode (1 ms rectangular pulse) using a constant current isolator (SS-102J; Nihon Koden Co., Ltd., Tokyo, Japan) coupled with an electrical stimulator (SEN7203; Nihon Koden Co., Ltd., Tokyo, Japan). An anode was placed above the patella, and a ball cathode was placed at the popliteal fossa. The H-reflex and M-wave responses were measured as peak-to-peak amplitudes of the non-rectified reflex. At the beginning of the experiment, the maximum amplitudes of H-reflex (Hmax) and M-wave (Mmax) were recorded in all participants. As the sensitivity of the H-reflex to facilitatory or inhibitory conditioning inputs was changed by the test H-reflex size, the size of the control SOL H-reflex was adjusted to 20–30% of Mmax in all conditions (Crone et al., 1990). Ten conditioned and ten unconditioned H-reflexes were recorded at each conditioning-test interval (C-T intervals), and the conditioned H-reflex amplitude was expressed as a percentage of the unconditioned H-reflex amplitude.

### Presynaptic Inhibition and Reciprocal Ia Inhibition

The H-reflex condition-test paradigm has been utilized to investigate presynaptic inhibition and reciprocal Ia inhibition in human subjects (Knikou, 2008).

The amount of presynaptic inhibition was determined from long latency (C-T intervals of 6–30 ms) suppression of the SOL H-reflexes, by conditioning stimuli to the common peroneal nerve (CPN) that innervates the TA muscle. This long-lasting SOL H-reflex suppression, which is called D1 inhibition (Mizuno et al., 1971), is correlated with the presynaptic inhibition of monosynaptic reflexes observed in animal studies in terms of the onset latency and slowly developing manner of H-reflex suppression (Eccles et al., 1962). The CPN was stimulated through a bipolar stimulation electrode (1 ms rectangular pulse) placed distal to the head of the fibula. The electrode was carefully positioned to avoid activating the peroneus muscles, and TA M-waves were monitored to ensure constancy of stimulation throughout the experiment. The intensities of the conditioning stimulus was set to just above the motor threshold (MT) intensity of the TA muscle (1.1 × MT). The CPN was stimulated with a train of three single pulses (1 ms rectangular pulse) at 333 Hz. The time interval between CPN stimulation (last shock of a train of three shocks) and test stimulation was kept constant at 15 ms. Conditioned and unconditioned H-reflexes were randomly evoked at 0.33 Hz.

The amount of reciprocal Ia inhibition was determined from a short latency (C-T interval of 2–3 ms) suppression of the SOL H-reflex by a conditioning stimulus to the CPN. This H-reflex suppression is believed to reflect reciprocal Ia inhibition (Crone et al., 1987), as the onset latency and response threshold (e.g., strength of conditioning stimuli) of the H-reflex suppression is comparable with the future of reciprocal Ia inhibition in cats (Eccles et al., 1956). The intensity of the conditioning stimulus was set to just above the MT intensity of the TA muscle (1.1 × MT). The CPN stimulus preceded the test stimulus at C-T intervals of 2 and 3 ms. The interval that produced the largest inhibition (either 2 or 3 ms) was used throughout the experiment. Conditioned and unconditioned H-reflexes were randomly evoked at 0.33 Hz.

### Transcranial Magnetic Stimulation (TMS)

A previous study has shown that motor cortical stimulation significantly decreases the D1 inhibition of SOL Ia afferents when applied 5–10 ms before the CPN stimulation (Meunier and Pierrot-Deseilligny, 1998). In these interstimulus intervals, cortical conditioning volleys reach the S1 spinal levels before the arriving CPN conditioning volleys, as conduction time from the stimulation site to the S1 spinal levels is approximately the same for the cortical and CPN stimulation (Herdmann et al., 1991; Meunier et al., 1994). Therefore, it is assumed that corticospinal descending inputs would produce the depressive effect on PAD interneurons in the lumber spinal cord (Rudomín et al., 1983). Moreover, it has been demonstrated that a conditioning TMS produces short and long latency facilitation of the H-reflex, which are considered to be due to monosynaptic and polysynaptic connections from corticospinal neurons (Nielsen and Petersen, 1995), respectively. Therefore, it is conceivable that these facilitation effects indirectly reflect the excitability of the corticospinal tract. In this study, we investigated the effects of corticospinal descending inputs on the presynaptic inhibitory pathway using TMS conditioning techniques.

Magnetic stimulation was delivered to the primary motor cortex (M1) through a double-cone coil connected to a magnetic stimulator (model 200; Magstim, Whitland, UK). The coil was placed on the scalp to induce a posterior-anterior current flow in the left M1. An optimal stimulus position for evoking motor evoked potentials (MEPs) in the right SOL muscle with a weak contraction was assessed by moving a coil around the leg motor area and was determined as the site where TMS at a slightly suprathreshold intensity regularly produced the largest MEPs. This position was marked with a pen on a swimming cap worn by the subject. Active MT was defined as the minimal stimulus intensity required to induce MEPs of at least 200 μV in three of five trials in the SOL muscle. While measuring the active MT, special care was given to maintain constant EMG activity levels of the SOL muscle (range, 100–150 μV). The intensity of conditioning TMS was set to 80–90% of the active MT, so that they had minor facilitation effects on the test H-reflex in a resting condition. To examine the effect of corticospinal descending inputs on the presynaptic inhibitory pathway, we compared the amount of D1 inhibition in the absence and presence of TMS. The time interval between TMS and CPN stimulation was set at 5 ms. Moreover, to study TMS conditioning effects on the test H-reflex, the amplitude of TMS conditioning H-reflex was evaluated at C-T intervals of 26 ms. For measurement of short latency facilitation effects, the intervals between conditioned TMS stimulation and H-reflex stimulation was set at −3, −2, and −1 ms. The C-T interval that produced the first facilitation effects on the H-reflex was regarded as a suitable C-T interval, and was used throughout the experiment. Negative C-T intervals indicated that the conditioned stimulus was applied after the test stimulation. Conditioned and unconditioned H-reflexes were randomly evoked at 0.2 Hz.

### Visuomotor Task

**Figure 1:**
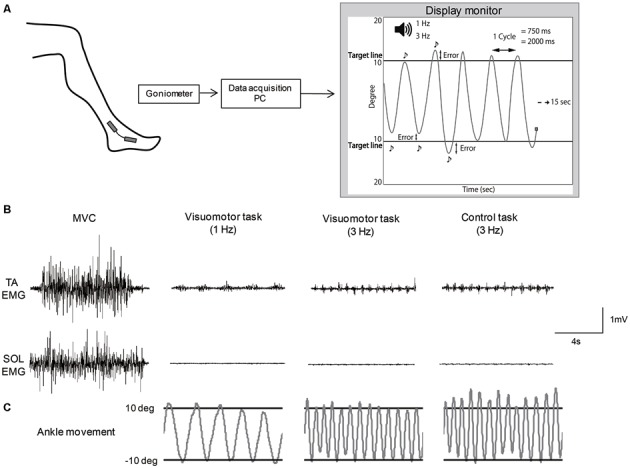
**Schematic of the motor tasks. (A)** Experimental set-up. **(B)** Example of raw EMG activity of tibialis anterior (TA) muscle and soleus (SOL) muscles during isometric maximum voluntary contraction (MVC) and each motor task. **(C)** Example of the angle joint movement during each motor task, which corresponded to electromyography (EMG) activity of TA and SOL muscles.

A custom made PC program (Labview 2012; National Instruments Co., Tokyo, Japan) was used to set up a visuomotor task. The subjects were allowed free movement of the ankle joint when performing the motor task. For a visuomotor task, subjects repeatedly moved their ankle between target lines according to auditory beep sounds delivered at a frequency of 1 Hz or 3 Hz (Figure 1A). In this task, subjects required precise control of joint movement. The movement speed was defined as the time of one cycle of ankle movement; slow movement speed was set to 2000 ms, and fast movement speed was set to 750 ms. The target lines representing the point of 10° angle of ankle dorsiflexion and of 10° angle of plantarflexion from the neutral position were displayed on a monitor (26-inch size), which was set at approximately 1 m in front of the subjects. The subjects were instructed to execute the ankle movement as precisely as possible between the target lines. Ankle angular displacements were measured with a goniometer (SG 100; Biometrics Ltd., Newport, UK) that was mounted on the lateral side of the leg (located at the fifth metatarsal and the fibula) and the goniometer signals were amplified by an amplifier (model 6L01; NEC San-ei Co. Ltd). The signals were recorded on a PC at a sample rate of 100 Hz via an A/D converter (USB6212; National Instruments Co.), and also displayed as a cursor with line trajectory on the display monitor, to control ankle movement. The cursor automatically moved from the left to the right at 15 s. Also, during ankle plantarflexion, the cursor moved to the top of the screen, whereas during dorsiflexion, the cursor moved to the bottom of the screen. A single trial example of raw EMG activity of the TA and SOL muscle and ankle movement during isometric maximum voluntary contraction (MVC) and each motor task is shown in Figures 1B,C. MVC was performed before the motor task and measured by pushing against a foot plate or pulling against a non-elastic band which was secured around the foot plate. The subjects performed the motor task for 20 min. The task session consisted of six blocks with five trials. In order to minimize muscle fatigue, 15 s resting periods occurred between trials, and each block was separated by 1 min. All subjects were familiarized with the motor task before starting the task session. Motor performance was quantified by the difference, subtracting from the actual degree and target degree (10° angle of dorsi- and planter-flexion) at each inflection point (Figure 1A). The value of the difference was defined as an error and averaged for each trial. As the number of inflection points was different between the slow movement speed condition and fast movement speed condition, the average value was determined from six cycles in the slow and 16 cycles in the fast movement condition at each trial, respectively, which matched the task performance time (12 s). The performance data were averaged for each block (mean error) and normalized by the value of the first block in order to confirm the rate of change in each task condition.

#### Experiment 1

Twenty-seven subjects participated in Experiment 1. They were randomly allocated to three different groups: slow speed group (*n* = 9), fast speed group (*n* = 9), and control group (*n* = 9).

The subjects who were assigned to slow and fast speed groups performed a visuomotor task, and the control group subjects performed a non-visuomotor task (control task) for 20 min. For the non-visuomotor task, the control group subjects repeatedly moved their ankle according to auditory beep sounds delivered at a frequency of 3 Hz (fast movement speed) without visual feedback of ankle movement. In this task, the subjects were not required precise control of joint movement. Other task procedures used the same visuomotor task.

We measured the amount of D1 inhibition and reciprocal Ia inhibition before (pre), 5 min after (post 5), 15 min after (post 15) and 30 min after (post 30) the task sessions. We also tested the ratio of Hmax vs. Mmax (Hmax/Mmax), which was used as an indicator of motor neuron pool excitability, before and immediately after the task session. Hmax and Mmax were evoked every 3 s and calculated from the average of five Hmax and five Mmax values.

#### Experiment 2

Sixteen subjects participated in Experiment 2. Five of the sixteen subjects also took part in Experiment 1, and the time between each experiment was at least 2 months. In Experiment 2, eight subjects performed the visuomotor task (visuomotor group) in the fast movement speed condition (3 Hz), and eight subjects performed the control task (non-visuomotor group). Task procedures were the same for the Experiment 1 protocol.

The amount of D1 inhibition, the amount of TMS conditioned D1 inhibition, the amplitude of TMS conditioned test H-reflex, the amplitude of TMS conditioned H-reflex amplitude at short facilitation phase, and Hmax/Mmax were measured before and after the task sessions.

### Statistical Analyses

The baseline characteristics of groups (age, SOL Mmax, Hmax/Mmax, active MT, and stimulus intensity of conditioning TMS) were analyzed using the unpaired *t*-test or one-way analysis of variance (ANOVA). The performance data compared among the task sessions used the one-way repeated measure of ANOVA for each group. Test H-reflex size was compared using two-way repeated measures of ANOVA with the factors “time” and “group.” In the Experiment 1 protocol, two-way repeated measures of ANOVA with the factors “time” and “group” were used to evaluate the effects of movement speed of the visuomotor task on the D1 inhibition and reciprocal Ia inhibition. In addition, the amounts of D1 inhibition and reciprocal Ia inhibition were compared using one-way repeated measure of ANOVA in the control group. In the Experiment 2 protocol, the amount of D1 inhibition, the amount of TMS conditioned D1 inhibition, the difference in the amount of D1 inhibition in the absence and presence of TMS, the amplitude of TMS conditioned test H-reflex, and the amplitude of TMS conditioned H-reflex at short latency facilitation phase were analyzed by two-way repeated measures of ANOVA with factors “time” and “group.” For multiple comparisons, if significant effects were detected, the Bonferroni *post hoc* test was used. Mauchley’s test was used to examine for sphericity. The Greenhouse-Geisser correction was used for non-spherical data. The amount of D1 inhibition was compared with the amount of TMS conditioned D1 inhibition within the group, using the paired *t*-test. The amplitude of TMS conditioned H-reflex at short latency facilitation phase was also compared with the unconditioned H-reflex, using the one-sample paired *t-test*. Moreover, in both experiments, the differences in the Hmax/Mmax and SOL Mmax were compared between before and after the task within the group, using the paired *t-test*. *P* values of < 0.05 were considered significant in all statistical analyses. Data were analyzed using SPSS version 22 software (IBM SPSS, IBM Japan, Ltd., Tokyo, Japan). The data values are presented as the means ± standard error of the mean (SEM).

## Results

Baseline characteristics of subjects among groups were well-matched in the Experiment 1 and the Experiment 2 groups (Table 1), and there were no significant differences between all baseline measures (age: *F*_2,24_ = 0.32, *P* = 0.73 in the Experiment 1 group, *t*_14_ = 1.41, *P* = 0.18 in the Experiment 2 group; SOL Mmax: *F*_2,24_ = 0.13, *P* = 0.87 in the Experiment 1 group, *t*_14_ = 0.69, *P* = 0.50 in the Experiment 2 group; Hmax/Mmax: *F*_2,24_ = 0.36, *P* = 0.69 in the Experiment 1 group, *t*_14_ = 0.06, *P* = 0.94 in the Experiment 2 group; active MT: *t*_14_ = 0.41, *P* = 0.68; Stimulation intensity: *t*_14_ = 0.83, *P* = 0.42).

### Changes in the Task Performance

**Figure 2:**
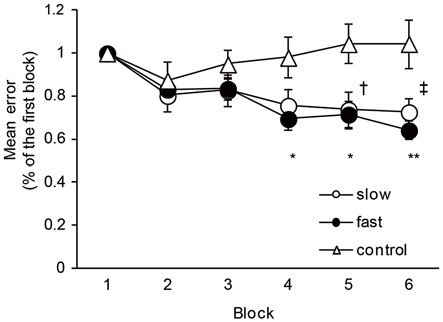
**The changes in the motor performance in slow-speed, fast-speed, and control groups.** The graph shows the time course of the changes in the motor performance in the slow, fast, and control groups. The ordinate shows mean error values normalized by the value of the first block. The abscissa shows each block. The dagger (†) represents significant difference (*P* < 0.05) between the first block and fifth block, and the double dagger (‡) represent significant difference (*P* < 0.01) between the first block and six block in the slow-speed group. The asterisks (*) represents significant difference (*P* < 0.05) between the first block and fourth and fifth blocks, and the double asterisks (**) represent significant difference (*P* < 0.01) between the first block and sixth block in the fast-speed group. Error bar indicates SEM.

The mean times of one cycle of ankle movement are shown in Table 2. The times almost matched the pre-setting times, indicating that the task movement speeds were well controlled throughout the experiment. Figure 2 shows the time course of the mean errors of motor tasks observed in the Experiment 1 group. A one-way repeated measures of ANOVA revealed a significant effect of blocks for slow and fast speed groups (Slow: *F*_5,40_ = 9.08, *P* < 0.01; Fast: *F*_5,40_ = 21.84, *P* < 0.01). In* post hoc* tests, a significant reduction in the mean errors was observed between the first block and five (*P* = 0.04) and sixth (*P* = 0.01) blocks in the slow speed group. Similarly, a significant reduction in the mean errors was observed between the first and fourth blocks (*P* = 0.01), between the first and fifth (*P* = 0.02) and sixth (*P* < 0.01) blocks in the fast-speed groups. These results indicated that the task performance was certainly improved across blocks in both groups. However, there were no significant differences in the mean errors across the blocks in the control group (*F*_5,40_ = 1.49, *P* = 0.21).

**Table 2:** **The mean times of one cycle of ankle movement (mean ± SEM)**.

	Time (ms)
Experiment 1
Slow speed group	1946.43 ± 5.97
Fast speed group	742.37 ± 2.36
Control group	733.77 ± 5.54
Experiment2
Visuomotor group	712.49 ± 19.73
Non-visuomotor group	708.83 ± 23.95

#### Experiment 1: Effects of Task Movement Speed on D1 Inhibition and Reciprocal Ia Inhibition

The mean amplitudes of the test H-reflex (% of Mmax) for all conditions are summarized in Table 3. The test H-reflex amplitude was almost the same size throughout the Experiment 1 procedures. There were no significant effects of “time” (D1 inhibition: *F*_3,72_ = 0.95, *P* = 0.41; reciprocal Ia inhibition: *F*_3,72_ = 1.48, *P* = 0.23) and “group” (D1 inhibition: *F*_2,24_ = 0.78, *P* = 0.46; reciprocal Ia inhibition: *F*_2,24_ = 0.35, *P* = 0.71) on the test H-reflex amplitude. Likewise, there was also no significant “time” × “group” interaction (D1 inhibition: *F*_3,72_ = 0.54, *P* = 0.78; reciprocal Ia inhibition: *F*_3,72_ = 1.18, *P* = 0.33).

**Table 3:** **Summary of test H-reflex amplitude in experiment 1 group (% of Mmax: mean ± SEM)**.

	D1 inhibition				Reciprocal Ia inhibition			
	Pre	Post 5	Post 15	Post 30	Pre	Post 5	Post 15	Post 30
Slow speed group	26.79 ± 0.85	25.27 ± 1.04	27.11 ± 1.07	25.28 ± 0.56	25.17 ± 0.60	25.69 ± 0.85	25.83 ± 1.01	25.40 ± 0.78
Fast speed group	25.76 ± 0.92	25.31 ± 1.12	24.35 ± 1.47	25.19 ± 0.85	25.43 ± 0.97	25.59 ± 1.05	23.34 ± 0.8	25.19 ± 0.85
Control group	25.99 ± 0.92	25.16 ± 0.91	25.15 ± 0.65	24.54 ± 1.16	25.37 ± 0.92	25.97 ± 0.74	24.67 ± 0.68	23.71 ± 1.09

*The values show the average of test H-reflex amplitudes calculated from the mean amplitude of test H-reflex in all subjects. Mmax, maximum amplitude of M-wave; Pre, before the task sessions; Post 5, 5 min after the task sessions; Post 15, 15 min after the task sessions; Post 30, 30 min after the task sessions*.

### Visuomotor Task

**Figure 3:**
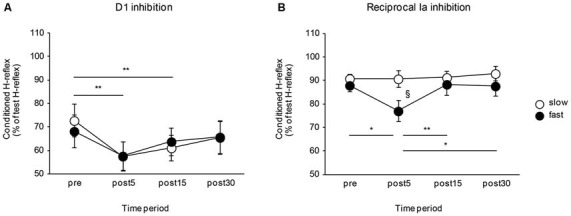
**Effects of the movement speed of visuomotor task on the D1 inhibition and reciprocal Ia inhibition.** The graphs show the mean values of the D1 inhibition **(A)** and reciprocal Ia inhibition **(B)** in the slow- and fast-speed groups. The ordinate indicates the conditioned H-reflex amplitude expressed as a percentage of the test H-reflex amplitude. The abscissa shows the times at which measurements were taken [before (pre), 5 min after (post 5), 15 min after (post 15), and 30 min after (post 30) the visuomotor task]. Open circles represent the slow-speed group and closed circles represent the fast-speed group. Values below 100% indicate inhibition and values above 100% indicate facilitation. The asterisks (*) and the double asterisks (**) represent significant differences (*P < 0.05) and (**P < 0.01), respectively. The section marked (§) represents a significant difference (*P* < 0.05) between slow- and fast-speed group in the post 5 time period. Error bar indicates SEM.

The effects of the movement speed of a visuomotor task on the D1 inhibition and reciprocal Ia inhibition are shown in Figure 3. The D1 inhibition increased after visuomotor task irrespective of task movement speed. Meanwhile, the reciprocal Ia inhibition was affected by the movement speed of the visuomotor task and only increased after performing the visuomotor task in the fast movement speed condition. A two-way repeated measures of ANOVA for D1 inhibition showed a significant effect of the “time” (*F*_2.06, 32.99_ = 14.84, *P* < 0.01), but not of the “group” (*F*_1,16_ < 0.01, *P* = 0.97). There was no significant “time” × “group” interaction (*F*_2.06, 32.99_ = 1.16, *P* = 0.33). *Post hoc* analysis of the “time” factor indicated that compared to pre, the amount of D1 inhibition was significantly increased in post 5 min (*P* < 0.01) and in post 15 min (*P* < 0.01). A two-way repeated measures of ANOVA for reciprocal Ia inhibition showed a significant effect of the “time” (*F*_3,48_ = 4.82, *P* < 0.01), but not of the “group” (*F*_1,16_ = 2.01, *P* = 0.18). Moreover, there were also significant “time” × “group” interactions (*F*_3,48_ = 3.49, *P* = 0.02). *Post hoc* analysis indicated that, in the fast-speed group, the amount of reciprocal inhibition was significantly increased in post 5 min—there were significant difference between pre and post 5 min (*P* = 0.01), post 5 min and post 15 min (*P* < 0.01), and post 5 min and post 30 min (*P* = 0.01). The amount of reciprocal Ia inhibition was also significantly different between slow and fast speed groups in the post 5 min time period (*P* = 0.03).

The mean SOL Mmax after the motor task was 13.86 ± 1.18 mV in the slow-speed group and 14.84 ± 2.09 mV in the fast-speed group. There were no significant differences in the SOL Mmax between pre and post in both groups (*t*_8_ = 1.22, *P* = 0.26 in slow-speed group; *t*_8_ = 0.40, *P* = 0.69 in fast-speed group). The mean Hmax/Mmax after the motor tasks was 0.51 ± 0.07 in the slow-speed group and 0.58 ± 0.07 in the fast-speed group. There were also no significant differences in the Hmax/Mmax between pre and post in both groups (*t*_8_ = 1.45, *P* = 0.19 in the slow-speed group; *t*_8_ = 1.07, *P* = 0.32 in the fast-speed group).

### Control Task

**Figure 4:**
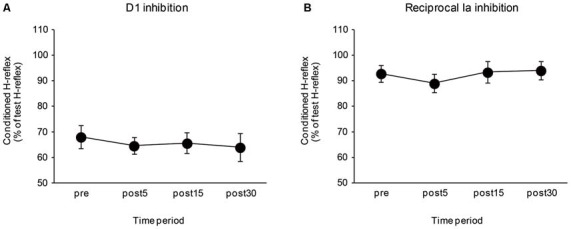
**Effects of the control task on the D1 inhibition and reciprocal Ia inhibition.** The graphs show the mean values of the D1 inhibition **(A)** and reciprocal Ia inhibition **(B)** in the control group. The ordinate shows the conditioned H-reflex amplitude expressed as a percentage of the test H-reflex amplitude. The abscissa shows the time at which measurements were taken [before (pre), 5 min after (post 5), 15 min after (post 15), and 30 min after (post 30) the visuomotor task]. Values below 100% indicate inhibition and values above 100% indicate facilitation. Error bar indicates SEM.

The effects of the control task on the D1 inhibition and reciprocal Ia inhibition are shown in Figure 4. There were no significant differences in the amount of D1 inhibition (*F*_3,24_ = 0.64, *P* = 0.57) and reciprocal Ia inhibition (*F*_3,24_ = 1.10, *P* = 0.37). The mean SOL Mmax after the control task was 14.02 ± 1.33 mV and the mean Hmax/Mmax after the control task was 0.55 ± 0.05. There were also no significant changes in the SOL Mmax (*t*_8_ = 0.08, *P* = 0.94), and Hmax/Mmax (*t*_8_ = 1.66, *P* = 0.14) following the control task.

#### Experiment 2: Effect of Corticospinal Descending Inputs on the Presynaptic Inhibitory Pathway

The mean amplitudes of the test H-reflex (% of Mmax) for all conditions are summarized in Table 4. The test H-reflex amplitude was almost the same size throughout the Experiment 2 procedures. There were no significant effects of “time” (D1 inhibition: *F*_1,14_ = 0.39, *P* = 0.54; TMS conditioned D1 inhibition: *F*_1,14_ = 4.07, *P* = 0.06; TMS conditioned H-reflex at short latency facilitation phase: *F*_1,14_ = 3.85, *P* = 0.07) and “group” (D1 inhibition: *F*_1,14_ = 0.54, *P* = 0.47; TMS conditioned D1 inhibition: *F*_1,14_ = 1.45, *P* = 0.25; TMS conditioned H-reflex at short latency facilitation phase: *F*_1,14_ = 2.03, *P* = 0.18) on the test H-reflex amplitude. There was also no significant “time” × “group” interaction (D1 inhibition: *F*_1,14_ = 0.19, *P* = 0.67; TMS conditioned D1 inhibition: *F*_1,14_ = 0.31, *P* = 0.59; TMS conditioned H-reflex at short latency facilitation phase: *F*_1,14_ = 0.55, *P* = 0.47).

**Table 4:** **Summary of test H-reflex amplitude in experiment 2 group (% of Mmax: mean ± SEM)**.

	D1 inhibition		TMS conditioned D1 inhibition		TMS conditioned H-reflex (short latency facilitation)	
	Pre	Post	Pre	Post	Pre	Post
Visuomotor group	25.11 ± 0.94	24.98 ± 0.99	25.53 ± 1.38	24.72 ± 1.01	25.54 ± 0.81	24.12 ± 0.71
Non-visuomotor group	26.39 ± 1.16	25.68 ± 1.15	27.41 ± 0.48	25.99 ± 0.94	26.42 ± 0.54	25.77 ± 0.81

*The values show the average of test H-reflex amplitudes calculated from the mean amplitude of test H-reflex in all subjects. Mmax, maximum amplitude of M-wave; Pre, before the task sessions; Post,after the task sessions;TMS, transcranial magnetic stimulation*.

**Figure 5:**
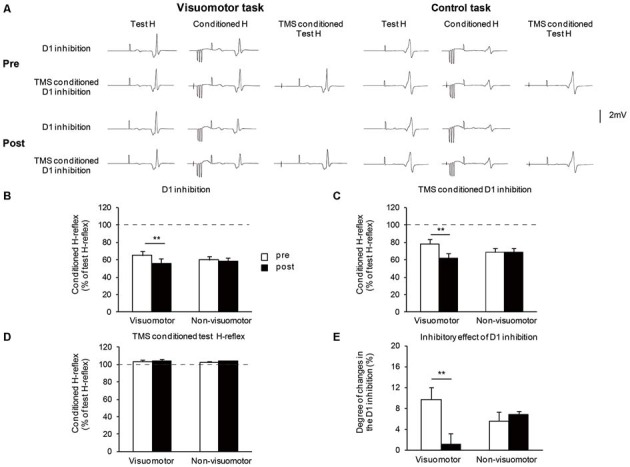
**The effect of visuomotor task and control task on the transcranial magnetic stimulation (TMS) conditioned inhibitory effects on the presynaptic inhibitory pathways. (A)** Typical averaged waveforms of H-reflexes (*n* = 10) in each stimulus condition were recorded from two representative subjects who performed a visuomotor task (left) or a control task (right). **(B–E)** The graphs show the mean values of the D1 inhibition **(B)**, TMS conditioned D1 inhibition **(C)**, TMS conditioned test H-reflex amplitude **(D)**, and the net difference in the amount of D1 inhibition **(E)**, in the visuomotor and non-visuomotor group. In **(B–D)**, the ordinate shows the conditioned H-reflex amplitude expressed as a percentage of the test H-reflex amplitude. The dashed line indicates the test H-reflex amplitude (100%). Values below 100% indicate inhibition and values above 100% indicate facilitation. In **(E)**, the ordinate shows the degree of changes in the D1 inhibition which is calculated by subtracting the minor facilitation effect of TMS conditioned test H-reflex amplitude (mean conditioned H-reflex—test H-reflex) from the changing amount of D1 inhibition in the absence and presence of TMS (difference between graph **B** and **C**, expressed as a percentage of the test H-reflex amplitude). Open and closed bars represent the time at which measurements were taken before (pre) and after (post) the motor task, respectively. The double asterisks (**) represent significant difference (**P < 0.01). Error bar indicates SEM.

Figure 5A shows the typical averaged waveforms (*n* = 10) of the control and conditioned H-reflexes induced by CPN stimulation and TMS stimulation, recorded from one representative subject. Figures 5B–D show the amount of D1 inhibition, the amount of TMS conditioned D1 inhibition, and TMS conditioned test H-reflex amplitude before and after the visuomotor task or control task, respectively. As the conditioning stimulation of TMS produced minor facilitation effects on the test H-reflex amplitude (Figure 5D), the net difference in the amount of D1 inhibition was calculated by subtracting this facilitation effect from the changing amount of D1 inhibition in the absence and presence of TMS [(graph C − graph B) − (100 − graph D)], which is shown in Figure 5E. A two-way repeated measures of ANOVA for D1 inhibition showed a significant effect of the “time” (*F*_1,14_ = 71.43, *P* < 0.01), but not of “group” (*F*_1,14_ = 0.03, *P* = 0.84). There was significant “time” × “group” interaction (*F*_1,14_ = 34.44, *P* < 0.01). *Post hoc* analysis indicated that compared to pre, in the visomotor group, the amount of D1 inhibition was significantly increased at post (*P* < 0.01). A two-way repeated measures of ANOVA for TMS conditioned D1 inhibition showed a significant effect of the “time” (*F*_1,14_ = 31.19, *P* < 0.01), but not of “group” (*F*_1,14_ = 0.02, *P* = 0.89). There was significant “time” × “group” interaction (*F*_1,14_ = 31.39, *P* < 0.01). *Post hoc* analysis indicated that compared to pre, in the visuomotor group, the amount of TMS conditioned D1 inhibition was significantly increased at post (*P* < 0.01). In the visuomotor group, the amount of TMS conditioned D1 inhibition was significantly greater than the amount of D1 inhibition at pre (*t*_7_ = 4.17 *P* < 0.01), but not at post (*t*_7_= 0.55, *P* = 0.59). In the non-visuomotor group, the amount of TMS conditioned D1 inhibition was significantly greater than the amount of D1 inhibition at the same period of time (pre: *t*_7_ = 3.56, *P* < 0.01; post: *t*_7_ = 9.16, *P* < 0.01). However, there were no significant effects of “time” (*F*_1,14_ = 0.52, *P* = 0.48) and “group” (*F*_1,14_ = 0.19, *P* = 0.66) on the TMS conditioned test H-reflex amplitude, and there was no significant “time” × “group” interaction (*F*_1,14_ < 0.01, *P* = 0.96). Moreover, a two-way repeated measures of ANOVA for the net difference in the amount of D1 inhibition showed a significant effect of “time” (*F*_1,14_ = 7.31, *P* = 0.02) but not of “group” (*F*_1,14_ = 0.22, *P* = 0.64). There was significant “time” × “group” interaction (*F*_1,14_ = 10.35, *P* < 0.01). *Post hoc* analysis indicated that compared to pre, in the visuomotor task group, the net difference in the amount of D1 inhibition was significantly decreased at post (*P* < 0.01). The inhibitory effect of D1 inhibition induced by TMS is decreased following the visuomotor task.

**Figure 6:**
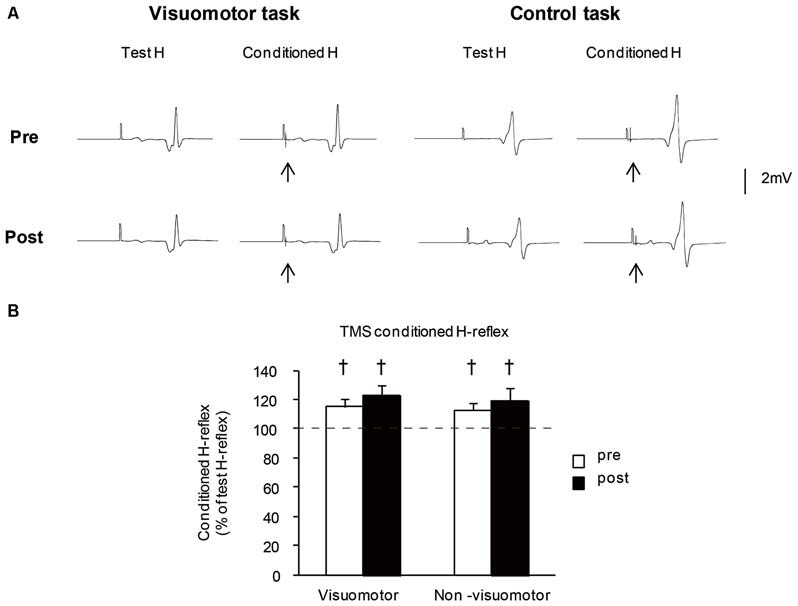
**The effect of visuomotor task and control task on the transcranial magnetic stimulation (TMS) conditioned H-reflex at short latency facilitation phase. (A)** Typical averaged waveforms of H-reflexes (*n* = 10) in each stimulus condition were recorded from two representative subjects who performed a visuomotor task (left) or a control task (right). The arrows indicate the artifact of TMS stimulation. The conditioning stimulation of TMS was applied after the test H-reflex stimulation. **(B)** The graphs show the mean values of the TMS conditioned H-reflex amplitude at short latency facilitation phase in the visuomotor and non-visuomotor group. The ordinate shows the conditioned H-reflex amplitude expressed as a percentage of the test H-reflex amplitude. Values below 100% indicate inhibition and values above 100% indicate facilitation. Open and closed bars represent the time at which measurements were taken before (pre) and after (post) the motor task, respectively. The daggers (†) represent significant differences (^†^*P* < 0.05) between conditioned H-reflex and baseline test H-reflex, which is shown by the dashed line. Error bar indicates SEM.

Figure 6A shows the typical averaged waveforms (*n* = 10) of the control and conditioned H-reflexes induced by TMS stimulation with the C-T intervals at short latency facilitation phase, recorded from one representative subject. The mean amplitudes of TMS conditioned H-reflex at short latency facilitation phase before and after the motor tasks are shown in Figure 6B. The amplitude of the TMS conditioned H-reflex was significantly larger than that of the unconditioned H-reflex in all conditions (pre: *t*_7_ = 22.21, *P* < 0.01 in the visuomotor group, *t*_7_ = 23.11, *P* < 0.01 in the non-visuomotor group; post: *t*_7_ = 17.77, *P* < 0.01 in the visuomotor group, *t*_7_ = 14.18, *P* < 0.01 in the non-visuomotor group). There were no significant effects of “time” (*F*_1,14_ = 1.47, *P* = 0.25) and “group” (*F*_1,14_ = 0.59, *P* = 0.46) on the TMS conditioned H-reflex amplitude, and there was also no significant “time” × “group” interaction (*F*_1,14_ = 0.03, *P* = 0.87). The short latency facilitation effects on the H-reflex amplitude did not show any significant difference between pre and post in both groups.

Following the motor tasks, the mean SOL Mmax was 16.68 ± 1.99 mV in the visuomotor group and 14.44 ± 1.38 mV in the non-visuomotor motor task group, and the mean Hmax/Mmax was 0.45 ± 0.04 in the visuomotor group and 0.49 ± 0.06 in the non-visuomotor group. There were no significant changes in the SOL Mmax (*t*_7_ = 0.99, *P* = 0.35 in the visuomotor group; *t*_7_ = 0.96, *P* = 0.37 in the non-visual group), and Hmax/Mmax (*t*_7_ = 2.10, *P* = 0.07 in the visuomotor group; *t*_7_ = 0.29, *P* = 0.78 in the non-visual group) following the control task.

## Discussion

The main findings of our study suggest that: (i) the amount of presynaptic inhibition is increased after visuomotor tasks irrespective of task movement speed; (ii) changes in the reciprocal Ia inhibition are affected by task movement speed, and are increased in fast movement speed conditions, but unchanged in slow movement speed conditions; (iii) non-visuomotor tasks do not induce any changes in presynaptic inhibition and reciprocal Ia inhibition; and (iv) TMS conditioned inhibitory effects on the presynaptic inhibitory pathway are changed following visuomotor tasks.

### Consideration of the Muscle Fatigue Effects

Because muscle fatigue enhances central excitability at the supraspinal levels and changes the presynaptic inhibition (Duchateau and Hainaut, 1993), it is conceivable that our results might be attributed to muscle fatigue. In human studies, muscle responses evoked by supramaximal peripheral nerve stimulation have been used as the index of muscle fatigue following exercise or electrical stimulation (Cupido et al., 1996; Lentz and Nielsen, 2002). In this study, we did not find any significant changes in the SOL Mmax before and after the motor task in any task conditions. The lack of changes in the Mmax suggests that muscle fatigue did not take place after the motor tasks. Moreover, the changes in the spinal neural circuits were only observed after visuomotor tasks, although demanding muscle activity levels were almost the same for the control task group (Figure 1B). Therefore, we argue that the observed modulations of presynaptic inhibition and reciprocal Ia inhibition are not caused by muscle fatigue.

### Methodological Consideration Associated with TMS Conditioning Techniques

We investigated the effect of corticospinal descending inputs on the presynaptic inhibitory pathway using TMS conditioning techniques. It has been demonstrated that conditioning TMS produces long latency facilitation (observed around 10–20 ms CT intervals) of the H-reflex (Nielsen et al., 1993). As the sensitivity of the H-reflex to the conditioning inputs depends on its size (Crone et al., 1990), the difference in the test H-reflex size is likely to affect the changes in D1 inhibition. Thus, to avoid this test size effect, we adjusted TMS stimulus intensity to evoke minor facilitation on the test H-reflex when TMS was given alone. Moreover, we observed that TMS conditioned H-reflex amplitude was not changed before and after the motor task, suggesting that test size effects could be negligible. By contrast, it is possible that the decrement of stimulation intensity might be inadequate to produce corticospinal descending volleys to the spinal cord. However, in this study, we confirmed the short latency facilitation effect on the H-reflex amplitude in the adjusted stimulation intensity. Therefore, it is reasonable to infer that corticospinal descending inputs induced by TMS would reach the spinal cord. Taking these results into account, we considered that our procedures for measuring the effect of corticospinal descending inputs on the presynaptic inhibitory pathway were appropriate.

### Effects of Task Movement Speed on Presynaptic Inhibition

In the present study, we observed that the presynaptic inhibition was increased following a visuomotor task irrespective of task movement speed, but was unchanged by a non-visuomotor task. Moreover, we also observed the improvement of the task performance among the trials in the slow and fast movement speed conditions. The increase in the presynaptic inhibition of SOL Ia afferent terminals following a visuomotor task was consistent with the results of a previous investigation which demonstrated that presynaptic inhibition was increased after visuomotor tracking tasks involving alternating ankle movement (Perez et al., 2005). Presynaptic inhibition of sensory signals has been shown to be correlated with precise control of limb movement as excessive proprioceptive inputs arising from muscle spindle afferents would produce limb oscillation (Fink et al., 2014). Thus, because muscle spindle response increases with increasing the velocity of muscle stretch (Popple and Bowman, 1970; Bosco and Poppele, 1999), we hypothesize that the presynaptic inhibition will be increased following motor training-performed at a fast movement speed, but not in a slow movement state. However, contrary to our hypothesis, the speed-dependent modulation of presynaptic inhibition was not observed in this study. This result suggests that the changes in presynaptic inhibition have little to do with the task movement speed. Previous studies have shown that the presynaptic inhibition is decreased after performing visoumotor force tracking tasks with thumb and index finger in isometric condition (Roche et al., 2011) or after isometric strength training on the ankle dorsiflexor muscles (Jessop et al., 2013). Although the effect of the motor task on presynaptic inhibition may differ between lower and upper limbs, the fact that the presynaptic inhibition was increased following only the visuomotor tasks involving dynamic joint movement indicates that descending inputs from supraspinal centers for controlling joint movement might be one of the essential factors to induce the potentiation of the presynaptic inhibition. It has been demonstrated that interneurons mediating presynaptic inhibition are controlled by supraspinal centers (Jankowska, 1992), and that the stimulation of the corticospinal tract decreases PAD in muscle afferent lower limbs (Rudomín, 1990; Meunier and Pierrot-Deseilligny, 1998). Therefore, we speculate that the changes in the presynaptic inhibition observed herein probably result from modifications of the interneurons interposed in the presynaptic inhibitory pathways, and supraspinal descending inputs play a major role in driving plastic changes in these interneurons.

To determine the influence of descending inputs on the changes in the presynaptic inhibition following a visuomotor task, we examined the TMS conditioning effect of presynaptic inhibition. We showed that the inhibitory effect of presynaptic inhibition induced by TMS was decreased following the visuomotor task, but not following the non-visuomotor task. This noninhibitory effect was not caused by the excitability changes in the corticospinal tract or motoneuron pool; the short latency facilitation effect of TMS stimulation on the SOL H-reflex and Hmax/Mmax were not changed by the visuomotor task or the non-visuomotor task. These results suggest that the changes in presynaptic inhibition observed in the present study are attributed to the activity changes in the interneurons constituting presynaptic inhibitory pathways. It has been shown that the corticospinal tract is essential both for producing spinal plastic changes and for maintaining its changes (Wolpaw, 2007). Although the detailed mechanisms related to the changes in presynaptic inhibition remain unknown, the increased presynaptic inhibition could conceivably be explained by reduced inhibition of the presynaptic inhibitory interneurons form the interneurons activated by the corticospinal tract. However, those interneurons receive inputs from a number of other sources such as rubrospinal fibers and cutaneous fibers (Rudomín et al., 1983) and may show plastic changes independent of corticospinal inputs.

Several studies have reported that H-reflex amplitude is decreased following skilled motor tasks (Perez et al., 2005; Mazzocchio et al., 2006; Lungu et al., 2010), and this modulation is explained by the changes in the presynaptic inhibition at the Ia afferent terminals. However, in this study we did not observe any changes in the H-reflex amplitude before and after the visuomotor task. The discrepancy between the changes in the H-reflex observed by our study and the previous studies is probably due to the difference in the motor task used in the study. In the previous studies, the muscle that assessed H-reflex was activated as an agonist during the task. By contrast, in this study, the subjects performed alternating ankle dorsal and plantarflexion movements against gravity so that the ankle dorsiflexion muscles acted as the prime movers for controlling ankle joints. Thus, the antagonist muscle was activated mainly, and the activation of the test muscle was minor (Figure 1B). Previous studies have reported that homosynaptic depression that reduces synaptic efficacy at the synapse between Ia afferent and motoneurons might be responsible for the decrease of the H-reflex after the skilled motor task (Mazzocchio et al., 2006; Meunier et al., 2007). Because homosynaptic depression has been suggested to be influenced by the pattern and magnitude of the incoming proprioceptive inputs (Meunier et al., 2007), the difference in the performing task might be attributed to the different results found in the present and previous studies.

### Effects of Task Movement Speed on Reciprocal Ia Inhibition

Training-related changes in the reciprocal Ia inhibition have been studied extensively in human subjects, and these studies have shown that there is also task dependency of the changes in the reciprocal Ia inhibition. For example, the facilitation effect of the reciprocal Ia inhibitory pathway at the onset of ankle dorsiflexion was increased following 4 weeks of explosive isometric dorsiflexion strength training (Geertsen et al., 2008). However, short-term isometric or isotonic strength training on ankle dorsal and planter flexor muscles decreased the reciprocal Ia inhibition (Jessop et al., 2013). Similar results were reported after performing force tracking tasks that required exerting an isometric force between the thumb and index finger (Roche et al., 2011). Moreover, the reciprocal Ia inhibition did not change after visuomotor tracking tasks involving alternating ankle movement (Perez et al., 2005). In our study, the amount of reciprocal Ia inhibition directed from TA to SOL was only increased when subjects performed a visuomotor task in the fast movement speed condition, but remained unchanged when the subjects performed a visuomotor task in the slow movement speed condition and the control task. These findings suggest that the changes in reciprocal Ia inhibition on the ankle muscles have something to do with the task movement speed.

The Ia inhibitory interneurons receive descending inputs from the supraspinal centers that are likely to influence the Ia inhibitory interneuron excitability (Jankowska et al., 1976; Kasai and Komiyama, 1988; Kubota et al., 2014). Therefore, it could be hypothesized that the increase of reciprocal Ia inhibition observed in our study is due to the modification of the excitability of Ia inhibitory interneurons induced by supraspinal descending inputs. Previous studies showed that the responses of corticomotoneuronal cells vary depending on type of movement (Fromm and Evarts, 1977), and that the activities of these cells are strong during controlled ramp-and-hold movement, compared with their activities during rapid alternating movement (Cheney and Fetz, 1980). However, our results also showed that performing the skilled motor task, itself, could not induce the increment in the strength of reciprocal Ia inhibition. Therefore, the enhancement of the reciprocal Ia inhibition could not be explained solely by the difference in the descending inputs from the corticospinal tract. The activation of Ia interneurons contributes to the hyperpolarization of target motoneurons (Geertsen et al., 2011), suggesting that Ia inhibitory interneurons play an important role in determining the coordination of intralimb flexor-extensor activity (Cowley and Schmidt, 1995). This may be because the reciprocal Ia inhibitory pathway needs to be facilitated in order to achieve alternating rapid movement. In support of this concept, the increase of reciprocal Ia inhibition was only observed following a visuomotor task in the fast movement speed condition. Taking these results into account, it is considered that both the central descending drive for controlling joint movement and task movement speed are important in driving activity changes in reciprocal Ia inhibition.

### Time Course Effects of the Visuomotor Task on Spinal Neural Circuits

The changes in the presynaptic inhibition and reciprocal Ia inhibition observed in this study were short lasting after the end of a visuomotor task; the increase in presynaptic inhibition lasted up to 15 min and the increase in reciprocal Ia inhibition lasted up to 5 min after the motor task. These temporary modifications of the spinal neural circuits almost consist with the previous reports (Perez et al., 2005; Roche et al., 2011). Previous studies have shown that long-lasting change in the spinal cord occurs gradually over weeks (after 10–12 sessions; Thompson et al., 2009, 2013). Short-lasting change is thought to reflect the change in supraspinal descending influence on the spinal cord, while long-lasting change is thought to reflect the spinal cord plasticity caused by the long-term continuation of the descending influence (Wolpaw, 2007). Therefore, we ended up with a speculation that the observed changes in the spinal neural circuits may reflect the early process of activity dependent plasticity in the spinal cord.

## Limitations

In the present study, all measurements were performed at rest, so it is difficult to see how supraspinal descending inputs are actually responsible for the observed changes in presynaptic inhibition and reciprocal Ia inhibition. Therefore, this should be taken into consideration when interpreting the present results.

## Conclusions and Implications

The results of this study suggest that the supraspinal descending inputs to the spinal cord for controlling joint movement are responsible for the changes in presynaptic inhibition, and that task movement speed is one of the critical factor for inducing activity changes in reciprocal Ia inhibition. These results indicate that spinal neural circuits are differentially modulated, dependent on motor tasks, for achieving the task demands. These task-dependent modulations might be related to the precise control of our limb movements.

## Author Contributions

SK, MH, YK, ST, and KF conceived and designed the research. SK, MH, and YK collected the data. SK analyzed data. All authors participated in the interpretation of the data. SK drafted the manuscript. All authors approved the final version of the manuscript submitted for publication.

## Conflict of Interest Statement

The authors declare that the research was conducted in the absence of any commercial or financial relationships that could be construed as a potential conflict of interest.

## Abbreviations

A/D: analog/digital
CPN: common peroneal nerve
C-T intervals: conditioning-test intervals
Hmax: maximum amplitude of H-reflex
M1: primary motor cortex
MEPs: motor evoked potentials
Mmax: maximum amplitude of M-wave
MT: motor threshold
PAD: primary afferent depolarization
SOL: soleus
TA: tibialis anterior
TMS: transcranial magnetic stimulation.
